# Flexural Strength of Polymethyl Methacrylate Repaired with Fiberglass

**Published:** 2017-07

**Authors:** Fariba Golbidi, Maryam Amini Pozveh

**Affiliations:** 1 Associate Professor, Dental Materials Research Center, Department of Prosthodontics, School of Dentistry, Isfahan University of Medical Sciences, Isfahan, Iran; 2 Assistant Professor, Department of Prosthodontics, School of Dentistry, Kashan University of Medical Sciences, Kashan, Iran

**Keywords:** Polymethyl Methacrylate, Denture Repair, Acrylic Resins, Fiberglass

## Abstract

**Objectives:**

The purpose of this experimental study was to discover a method to increase the strength of repaired polymethyl methacrylate (PMMA) samples.

**Materials and Methods:**

In this experimental study, 40 specimens with the dimensions of 65×10×2.5mm^3^ were fabricated using heat-curing acrylic resin. Sixteen specimens were repaired with fiberglass and self-curing PMMA, while 16 samples were repaired with self-curing PMMA. Eight specimens were left intact as the control group. Afterwards, the flexural strengths of the repaired and intact specimens were measured by three-point bending test in a universal testing machine. Data were analyzed with one-way analysis of variance (ANOVA) and Tukey’s HSD and LSD tests. The level of significance was set at P<0.05.

**Results:**

The mean flexural strength of the samples repaired with fiberglass was higher than that of the other repaired samples. However, the difference was statistically significant only with respect to the Meliodent group (P=0.008).

**Conclusions:**

Impregnated fiberglass could be used in the repair of denture bases to improve the flexural strength. In terms of the fracture site, it can be concluded that the lower flexural strength of the auto-polymerizing acryl compared to that of the heat-curing type was the main reason for the occurrence of fractures, rather than the weak bond between heat-curing and auto-polymerizing acrylic resins.

## INTRODUCTION

Polymethyl methacrylate (PMMA) has been the most commonly used material in denture base fabrication since 1940 [1]. Its favorable characteristics include acceptable aesthetics and ease of use and repair. However, PMMA has some unfavorable mechanical properties such as poor thermal conductivity [2], low flexural and fatigue strengths [3,4] and low impact strength [5]. Many efforts have been made to improve these mechanical properties including the addition of glass, aramid and nylon fibers [6], polyethylene fibers [7] and nano-components [8,9]. Low flexural strength causes acrylic denture to fracture due to powerful masticatory forces, ill-fitting denture bases, or improper occlusion. Fractures of acrylic denture are the main reason for denture repair [10,11]. Different materials are available for denture repair. The one most often used is auto-polymerizing acrylic resin. The flexural strength of auto-polymerizing acryl is lower than that of the heat-curing type; therefore, recurring fractures can be expected [12,13]. Several studies have tried to find methods to strengthen the repaired dentures through modification of the repair interface [14], surface treatment [15,16], metal fiber reinforcement [17] and fiberglass reinforcement [18–20]. Nowadays, fiberglass is one of the most favored fibers in dentistry because of proper aesthetics and acceptable bonding. Several studies have reported that addition of fiberglass has improved the flexural strength of PMMA [19,20]; however, some other studies have indicated that reinforcement with fiberglass has no significant effect on the flexural strength [21]. One study ascertained that impregnated fiberglass provided higher flexural strength than conventional fiberglass [22]. Incorporating fiberglass in PMMA repair could affect the fracture site of the PMMA. The aim of this study was to compare the strength of repaired denture base with and without the use of fiberglass. The null hypothesis was that the use of fiberglass has no effect on the flexural strength or the fracture site of the repaired acryl.

## MATERIALS AND METHODS

### Preparation of samples:

This experimental study was conducted at the dental materials laboratory of Isfahan University of Medical Sciences. The specimens were fabricated in accordance with the American Dental Association (ADA) Specification No. 12 for denture bases. The first four aluminum dies with the dimensions of 65×10×2.5mm^3^ were constructed and placed in a dental flask. After setting of the dental stone, the two pieces of the flask were separated, and the dies were removed.

Heat-curing acrylic resin (Meliodent, Bayer UK Ltd, Newbury, Berkshire UK) was prepared in accordance with the manufacturer’s instructions. The amount of 23.4g powder and 10ml monomer were mixed within 30 seconds. After 10 minutes, the mixture attained a doughy form and was placed in the flask. After placing a wet shell layer, the flask was closed and pressed at 1.5 bar pressure.

**Fig. 1:**
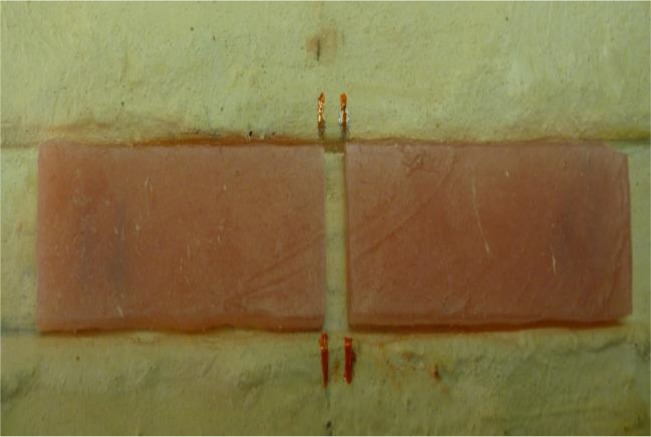
Sample to be repaired with auto-polymerizing acryl

**Fig. 2:**
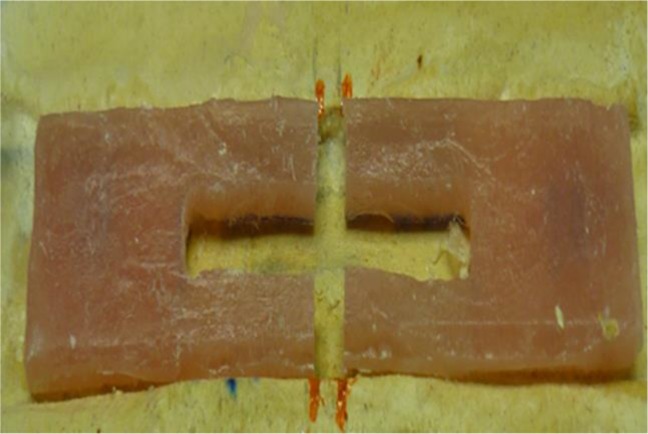
Sample to be repaired with auto-polymerizing acryl and fiberglass

Afterwards, the flask was opened, the shell layer was removed, the flask was closed, and 2 bar pressure was applied for 10 minutes. Then, the flask was entered into 15°C water in an automatic machine, which was turned on immediately. The flask was stored at 74°C for 1.5 hours and at 95°C for 1 hour. The machine was turned off automatically, and the flask was gradually cooled during 24 hours. Afterwards, the two pieces of the flask were separated gently through pulling the two pieces with hand force, and the specimens were removed without any stress. By following the above steps, 40 samples were made from heat-curing PMMA. Eight specimens were left intact as the control group. Sixteen specimens undergoing repair with auto-polymerizing acryl were divided into two equal segments with the use of a disc (Fig. 1). A track with the 30mm length and 3.5mm width was made at the center of the 16 specimens undergoing repair with auto-polymerizing acryl and fiberglass, and the specimens were cut at the midpoint using a disc (Fig. 2).

### Repairing the specimens:

A stone mold was prepared to hold the specimens. The specimens were kept steady during the repair and were removed without stress after the repair. A distance of 3mm was marked on the mold, and the two pieces of each specimen were fixed in this space. Eight specimens were repaired with Acropars auto-polymerizing acrylic resin (Marlic Medical Industries Co., Tehran, Iran), and 8 specimens were repaired with Meliodent auto-polymerizing acrylic resin (Bayer UK Ltd, Newbury, Berkshire UK) by using the sprinkle-on technique, in which the powder and monomer are sprinkled repeatedly layer by layer. After the repair, the specimens were immediately stored in a pressure pot (EWL Type 5415, Germany) for 10 minutes. Sixteen specimens were repaired with impregnated fiberglass (Angelus, Londrina, Brazil).

First, a layer of auto-polymerizing acrylic resin was sprinkled on the track, and then a 20mm-piece of impregnated fiberglass was placed on this layer and was covered with auto-polymerizing acryl (8 specimens were covered with Acropars acryl, and 8 specimens were covered with Meliodent acryl). The specimens were stored in the pressure pot for 10 minutes immediately after the repair.

All the steps were performed by the same technician. The specimens were stored in distilled water at 37°C for 50 hours. Afterwards, flexural strength was measured in a universal testing machine (Dartec HC 10, Stourbridge, UK) using 3-point bending test. The distance between the two bases of the machine was 50mm and the crosshead speed was 5mm/minute. The specimens were loaded to the fracture point. The flexural strength was then measured by using the following equation: S=3pl/2bd^2^, in which S is the flexural strength, p is the force at the fracture point, l is the distance between the two parts of the machine’s base, b is the specimen’s width, and d is the specimen’s thickness. Data were analyzed by one-way analysis of variance (ANOVA) and Tukey’s HSD and LSD tests. The level of significance was set at P<0.05.

## RESULTS

**Fig. 3:**
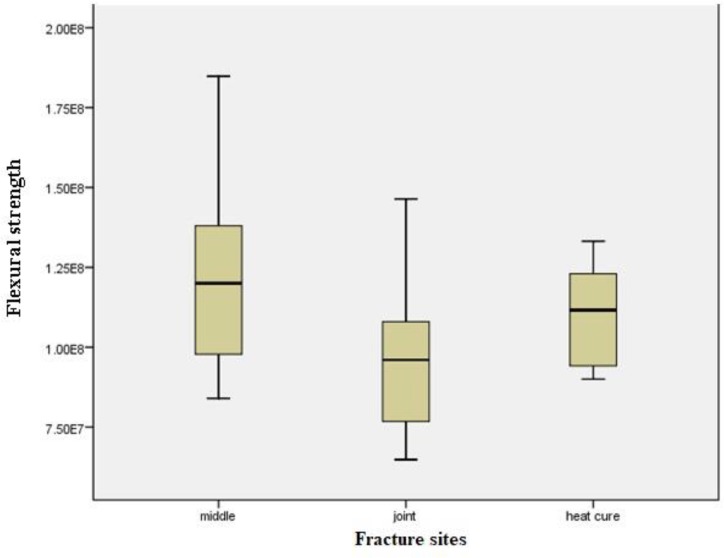
Boxplot of flexural strength according to the fracture site

The specimens which had been repaired with Meliodent auto-polymerizing acryl and fiberglass showed high flexural strength (126 MPa), whereas the specimens repaired with Acropars auto-polymerizing acryl without fiberglass exhibited low flexural strength (76.2 MPa). Table 1 presents the statistical components of the analysis. Regarding the homogeneity of variance, one-way ANOVA indicated that the differences between the groups were significant (P<0.001). Tukey’s HSD test, which was used to compare the mean flexural strength between the two groups, indicated that the flexural strengths of the groups repaired with Acropars acryl (P=0.001), and Acropars acryl plus fiberglass (P=0.03) were significantly lower than that of the control group. However, the groups repaired with Meliodent acryl (P=0.86), and Meliodent acryl plus fiberglass (P=0.85) exhibited no significant differences with the control group. The present study indicates that fiberglass has improved the flexural strength of the specimens repaired with Meliodent and Acropars acrylic resins; however, the difference was significant only with regard to the Meliodent group (Meliodent P=0.008, Acropars P=0.678). Although the flexural strength of Meliodent group was higher than that of Acropars specimens, the difference was not statistically significant (P=0.42). The specimens fractured at three different sites. 18.75% of the fractures occurred in the central part of the repaired specimens, 50% of the fractures occurred at the interface between auto-polymerizing and heat-curing acrylic resins, while 31.25% of the fractures were located within the heat-curing acryl. One-way ANOVA indicated that there was a significant correlation between the fracture site and flexural strength (P=0.04). Tukey’s LSD test showed that the fracture site was significantly correlated with the flexural strength at the central part of the samples and also at the interface between the repair site and heat-curing acryl (P=0.02). In specimens with lower flexural strength, the fractures appeared more frequently in the central part of the repaired specimens rather than at the interface. Figure 3 shows the correlation between the flexural strength and fracture site.

**Table 1.** Descriptive values of flexural strength

**Group**	**N**	**Mean (MPa)**	**Std. Deviation**	**Std. Error**	**Minimum (MPa)**	**Maximum (MPa)**
Control	8	117.30	12.87	4.55	103.20	141.60
Meliodent + Fiberglass	8	126.60	22.62	7.99	102.00	172.80
Meliodent	8	92.55	16.85	5.95	64.80	121.20
Acropars + Fiberglass	8	88.65	23.22	8.21	57.60	126.00
Acropars	8	76.20	16.34	5.77	52.80	96.00

## DISCUSSION

Investigations have shown that auto-polymerizing acrylic resin is the most frequently used material in denture repairs [21]. The flexural strength of auto-polymerizing acryl is lower than that of the heat-curing type; therefore, recurring fractures happen due to strength incongruity [12,13]. According to previous studies, the flexural strength of auto-polymerizing acryl is 18 to 81% lower than that of heat-curing acryl [12,13,23–25]. This was also confirmed by the present study, which indicated that the flexural strength of auto-polymerizing acryl was between 65% (Acropars) to 79% (Meliodent) of that of heat-curing acryl. To date, many studies have tried to improve the flexural strength of the repaired acryl to inhibit repeated fractures, through modifying the repair interface [14], surface treatment [15,16], or incorporating metal wire [18]. Golbidi and Mousavi [17] stated that the flexural strength of Acropars acrylic resin was significantly lower than that of Meliodent acryl, which contradicted our results. This difference can be attributed to the use of a pressure pot in the current study, which improved the flexural strength. Chemical adhesion occurs due to chemical bonding between the fiber and matrix. Pure fiberglass cannot establish this bond; therefore, it is necessary to apply silane coupling agent to fiberglass to establish a proper bond with the matrix, which improves the flexural strength of the composite resin [26]. In the present research, we used a composite resin infused with fiberglass, which was expected to improve the chemical bonding between the acryl and resin part of the infused fiberglass. Water can degrade the siloxane bond between the resin matrix and fiberglass [27], which causes hydrolytic instability in the sensitive glass components [28]; therefore, we placed the fiberglass at the depth of the specimen to keep it far from moisture. Reinforcement with fibers depends on the direction of every fiber. Unidirectional fibers improve strength only in one direction, whereas multidirectional fibers enhance the strength in all directions [20]. In the present study, braided fiberglass improved the flexural strength perpendicular to the force, which was significant with regard to the auto-polymerizing Meliodent acrylic resin. The results of the present study are in accordance with the studies by Polyzois et al [18], and Kostoulas et al [20], that reported enhancement in the flexural strength of acryl after the use of fiberglass, but are in contrast to the study by Minami et al [29], which indicated that fiberglass failed to improve the flexural strength. The mentioned study was performed under different conditions and with the woven type of fiberglass, whereas braided fiberglass was used in the present study. The fracture site of the repaired acryl is an important factor in reinforcement. Low flexural strength caused fractures at the central site of the samples instead of the interface between auto-polymerizing and heat-curing acrylic resins. Therefore, it can be concluded that low flexural strength of the auto-polymerizing acryl was the main reason for the recurrence of fractures in the repaired specimens, rather than the weak bond between auto-polymerizing and heat-curing acrylic resins. The present study is limited due to being performed under laboratory conditions, which are different from the oral environment. It is recommended to perform this study under dynamic conditions, including storage of the samples in saliva and/or application of mechanical and thermal cyclic stresses.

## CONCLUSION

The flexural strength of the repaired samples was lower than that of the intact samples, except for Meliodent samples repaired with impregnated braided fiberglass. The two types of auto-polymerizing acrylic resins (Acropars and Meliodent) had no significant differences in flexural strength. In terms of the fracture site, it can be concluded that lower flexural strength of the auto-polymerizing acryl compared to that of the heat-curing type is the main reason of fractures, rather than the weak bond between heat-curing and auto-polymerizing acrylic resins.
